# Spectrum Sensing Implemented with Improved Fluctuation-Based Dispersion Entropy and Machine Learning

**DOI:** 10.3390/e23121611

**Published:** 2021-11-30

**Authors:** Gianmarco Baldini, Jean-Marc Chareau, Fausto Bonavitacola

**Affiliations:** 1 European Commission, Joint Research Centre, 21027 Ispra, Italy; Jean-Marc.CHAREAU@ec.europa.eu; 2 Fincons, 20871 Vimercate, Italy; Fausto.Bonavitacola@ingpec.eu

**Keywords:** spectrum sensing, entropy, machine learning, signal processing

## Abstract

Spectrum sensing is an important function in radio frequency spectrum management and cognitive radio networks. Spectrum sensing is used by one wireless system (e.g., a secondary user) to detect the presence of a wireless service with higher priority (e.g., a primary user) with which it has to coexist in the radio frequency spectrum. If the wireless signal is detected, the second user system releases the given frequency to maintain the principle of not interfering. This paper proposes a machine learning implementation of spectrum sensing using the entropy measure as a feature vector. In the training phase, the information about the activity of the wireless service with higher priority is gathered, and the model is formed. In the classification phase, the wireless system compares the current sensing report to the created model to calculate the posterior probability and classify the sensing report into either the presence or absence of wireless service with higher priority. This paper proposes the novel application of the Fluctuation Dispersion Entropy (FDE) measure recently introduced in the research community as a feature vector to build the model and implement the classification. An improved implementation of the FDE (IFDE) is used to enhance the robustness to noise. IFDE is further enhanced with an adaptive method (AIFDE) to automatically select the hyper-parameter introduced in IFDE. Then, this paper combines the machine learning approach with the entropy measure approach, which are both recent developments in spectrum sensing research. The approach is compared to similar approaches in literature and the classical energy detection method using a generated radar signal data set with different conditions of SNR(dB) and fading conditions. The results show that the proposed approach is able to outperform the approaches from literature based on other entropy measures or the Energy Detector (ED) in a consistent way across different levels of SNR and fading conditions.

## 1. Introduction

This paper deals with the problem of spectrum sensing for coexistence of wireless services in the same radio frequency spectrum bands. The rapid development of wireless communication technologies has increased the need to use the radio frequency spectrum in an efficient way. One of the solutions proposed in research literature to enhance the utilization of radio frequency spectrum resources especially in frequency bands with low spectrum usage is to allow the coexistence of one or more wireless services. One example of this model is spectrum sharing where there is priority access for primary users (PU), but secondary users (SU) can still access the radio frequency spectrum in an opportunistic way if the PU signal is not present either in time or space. To determine the presence of the PU in the radio frequency spectrum, various techniques have been proposed in literature with spectrum sensing as one of the most common. Spectrum sensing is defined in [1] as “Spectrum sensing is the task of obtaining awareness about the spectrum usage and existence of primary users in a geographical area. This awareness can be obtained by using geolocation and database, by using beacons, or by local spectrum sensing at cognitive radios”. In this paper, we focus on the implementation of local spectrum sensing, which can be classified in blind spectrum sensing approaches, and knowledge-aided spectrum sensing approaches [2]. The blind spectrum sensing approaches require no prior knowledge of the PU signal characteristics, while the knowledge-aided spectrum sensing approaches require a full or partial prior information about the signal characteristics (e.g., modulation, data structure) of the PU signal. In this paper, we focus on a spectrum sensing approach to improve its generalization. The approach proposed in this paper is “almost blind” as we only use the duration of the PU signal to define the range of the hyper-parameter used in the approach, but we do not employ other information on the PU signal (e.g., modulation scheme or signal structure). One of the most common blind spectrum sensing approaches is based on the ED because of its simplicity and computing efficiency, but it is known not to be robust against the presence of noise [2].

The study presented in this paper proposes an implementation of spectrum sensing using Machine Learning (ML) algorithms. The synergy of these two research areas have received increased interest by the research community in recent years. ML is a way of programming computers to optimize a performance criterion using example data. ML has been increasingly used in the telecommunication network community for a variety of tasks including traffic prediction, traffic analysis, resource management, network security, and other tasks. A recent and extensive survey on the application of ML to telecommunication is provided in [3], but the scope of ML in this study is limited to spectrum sensing. In this context, ML creates a model of the PU signal directly from the collected data without prior knowledge of the modulation scheme of the signal and then uses the model to detect the presence or absence of the PU signal in the radio frequency spectrum. Then, the application of ML to spectrum sensing is based on the consideration that, since the task of determining the channel status is intrinsically a classification task, several authors have considered the use of ML models as inference tools. A survey on the application of ML and its extension to Deep Learning is provided in Section 2 of this paper, but it can be anticipated that this study focuses on a specific implementation of ML for spectrum sensing based on the application of entropy measures. A number of papers have applied entropy measures [4,5,6] to spectrum sensing because of its robustness to the presence of noise and because it reduces the dimensionality of the channel status evaluation problem. Spectrum sensing may involve the processing of a large number of samples (e.g., the digital representation of the signal in space), which can be reduced by applying feature extraction including the application of entropy measures. Then, the approach proposed in this paper combines the application of entropy measures with ML to the problem of spectrum sensing. In particular, the proposed approach uses the recently introduced Dispersion Entropy (DE) [7] and its variation FDE [8] instead of the Shannon entropy or Renyi entropy commonly used in literature on this specific problem (see Related work in Section 2). FDE has demonstrated a superior performance to Shannon entropy in various domains including the analysis of non-stationary electromagnetic signals in [9] (for radio frequency fingerprinting) and [10] (for analysis of electromagnetic discharge signals), but its application to spectrum sensing is non existent to the knowledge of the authors. In addition, this paper proposes an improved versions of the FDE to enhance the robustness to noise and fading effects. While most of the studies, which are reviewed and described in research literature, evaluate the application of entropy measures thorough simulations, this study evaluates the application of FDE and its improved variants described in the rest of this paper: Improved Fluctuation Dispersion Entropy (IFDE) and Adaptive Improved Fluctuation Dispersion Entropy (AIFDE) on a data set of generated radar signals which are supposed to coexist in the 5.9 GHz band with RLAN. The evaluation is performed for different values of Signal Noise Ratio (SNR) in dB and for different fading conditions. The results presented in the Results Section 5 show that the spectrum sensing implemented with FDE, IFDE, and AIFDE outperforms other entropy measures and the ED in a coherent manner across the different SNRs and fading conditions.

To summarize, this paper provides the following main contributions:

- The implementation of spectrum sensing based on the combination of entropy measures with ML algorithms.
- The novel application of FDE to the spectrum sensing problem.
- An improvement of the FDE and DE to make it more robust to the presence of noise.
- The evaluation of the proposed approach on a data set of real signals in different SNR and fading conditions.

The paper is composed by the following sections: Section 2 provides an overview of the related work. Section 3 describes the overall methodology, the materials used, the ML algorithms, and the related classification metrics. In particular, this section describes the generation of the signals used in the empirical evaluation. Section 4 describes at length the entropy measures, which have been used for the analysis with a particular focus on the FDE and its improved versions. Section 5 presents the results, where the performance of the different entropy measures and the baseline ED is given. Finally, Section 6 provides the conclusions to this paper and potential future developments.

## 2. Related Work

Research literature on spectrum sensing in the last 10–15 years is quite extensive, and it is not the intention of this section to report on all the possible implementations of spectrum sensing in literature. Extensive surveys on spectrum sensing are available in [1,11,12,13]. The focus of this section is to report on the application of entropy measures to spectrum sensing, on the application of ML to spectrum sensing and the combination of these two areas.

The rationale to apply entropy measures to this specific problem is that classical spectrum sensing techniques like the ED are particularly sensitive to the presence of noise while entropy measures can provide robust spectrum sensing implementation as in [4], where Shannon entropy in the frequency domain has been used. The reason why Shannon entropy was applied in the frequency domain (through a Discrete Fourier Transform) is because it is dependent on the signal power and is highly susceptible to noise uncertainty in the time domain. Therefore, the entropy is calculated in the frequency domain. In fact, this approach has been quite popular in literature since it has been applied to various spectrum sensing scenarios and cognitive networks typologies (e.g., cooperative sensing, in combination with cyclostationary detectors) like [14,15]. Previous works adopted the Shannon entropy measure in the time domain like [16] where a superior detection performance in comparison to ED and cyclostationary detection techniques is also demonstrated. All the papers cited until now are mostly based on simulations and none applies ML approaches for spectrum sensing. In addition, most of the cited papers use Shannon entropy as entropy measure. Some studies have also used other entropy measures. For example, the authors in [6] have used Renyi entropy (a generalization of Shannon entropy) in the frequency domain, and they have compared it with the ED showing the superior performance of the Renyi entropy based approach. On the other side, the authors of [6] do not use a ML approach as in this study. Another paper, which adopted Renyi and Shannon entropy in combination with the Parzen window, is [5], where the performance of the proposed approach was applied to simulated data. The results show that the approach outperforms the ED. In [5], the authors also do not use a ML approach. The Shannon entropy and the Renyi entropy are used in this study for comparison with the proposed approach based on FDE. We would like to highlight that the FDE is different from the entropy measures adopted in the previous papers because it is based on the distribution of the patterns in the signal in time rather than the distribution of the values of the signal. More details on the definition of the FDE are provided in Section 4. The review presented so far is mostly focused on non-cooperative spectrum sensing since this is the objective of this paper, but there are also a number of studies where entropy measures are used in a cooperative design where the wireless communication nodes cooperate among them to implement the spectrum sensing function. For example, the authors in [17] have applied Shannon entropy in a cooperative spectrum sensing design obtaining a higher detection performance than the ED. In another recent study [18], the authors have applied Shannon entropy measures to a cooperative spectrum sensing implementation using USRP equipment. The results confirm the superior detection performance on the application of the entropy measure in comparison to the ED.

From another point of view, there are many reported studies on the application of ML and Deep Learning (DL) to spectrum sensing. A recent and extensive survey on the application of ML and DL to spectrum sensing including wideband and/or cooperative spectrum sensing is presented in [19]. In the next paragraph, we focus on some relevant studies which may be related to this paper. In [20], the authors have compared different ML algorithms including Gaussian Mixture Model (GMM), Support Vector Machine (SVM), and K Nearest Neighbor (KNN) in a cooperative sensing scenario. The energy levels of the signal in space are used for the classification and the results show that the ML approach is able to outperform the classical cooperative sensing techniques. In a similar way, the authors in [21] have applied SVM to spectrum sensing using the energy levels as features in a cooperative spectrum sensing design. In [22], the authors present a new ML-based cooperative spectrum sensing (CSS) model, which utilizes the methods of user grouping to reduce cooperation overhead and effectively improve detection performance. The selected ML algorithm is the SVM. Cognitive radio users are properly grouped before the cooperative sensing process using energy data samples and an SVM model. Experimental results showed that the proposed algorithms achieved their intended function and outperformed conventional ML algorithms from literature in terms of security, energy consumption, and sensing efficiency.

A recent paper, which also applied ML to spectrum sensing, is [3], where different ML algorithms like multilayer perceptron, SVM, and Naive Bayes are applied to the energy levels of the channel (simulated data are used). The results show that SVM using a Gaussian kernel (a choice also adopted in this paper) outperforms the other algorithms. Entropy measures are not used in [3] and the previously cited papers, but the energy levels (either in the time or the frequency domain) are used. This study applies entropy measures rather than the energy levels of the signal in space for classification demonstrating that entropy measures provide a superior performance. On the other side, this study exploits the findings of the previously cited ML papers to select the appropriate ML algorithms (e.g., SVM, Decision Tree, KNN). Another recent paper [23] applies ML to multiband spectrum sensing where the challenge is to detect signals based on non-contiguous spectrum bands. Different machine learning algorithms (neural networks, expectation maximization and k-means) are applied to a multiband spectrum sensing technique for cognitive radios with k-means obtaining the best results in terms of detection performance and computing efficiency.

Recent papers have also started to apply DL to spectrum sensing. For example, the authors of [24] have applied deep learning to spectrum sensing with different types of noise using also real signals in addition to simulations. The application of deep learning is seen to outperform “shallow” ML algorithms like decision tree, SVM, and KNN. In [25], the authors apply deep Convolutional Neural Networks (CNN) to the spectrum sensing problem in a cooperative setting and using the spectral representation of the signal as input to the CNN. In [26], the authors apply DL in combination with spectrograms collected from the field measurements to investigate the radar coexistence problem which is similar to scenarios presented in this paper where radar signals are also evaluated. In both cases, DL is able to outperform ’shallow’ ML algorithms regarding the detection accuracy but at the cost of using considerable computing resources and time, which may be a problem in the context where spectrum sensing should operate with a very fast detection and reaction time and where the wireless devices may have limited computing capabilities. For this reason, this paper uses only ’shallow’ ML algorithms.

There are very few studies which combined ML and entropy measures in literature.

One very recent paper [27] (published in 2021) implements a similar study to this paper. The authors of [27] merge ML methods with the application of entropy measures. In the study, differential entropy is compared with energy detection methods, demonstrating to outperform them. As in the study presented in this paper, the evaluation is performed on real signals rather than a simulation. Contrary to this study, FDE is not used since differential entropy is derived from Shannon entropy.

## 3. Materials and Methods

### 3.1. Overall Methodology

**Figure 1:**
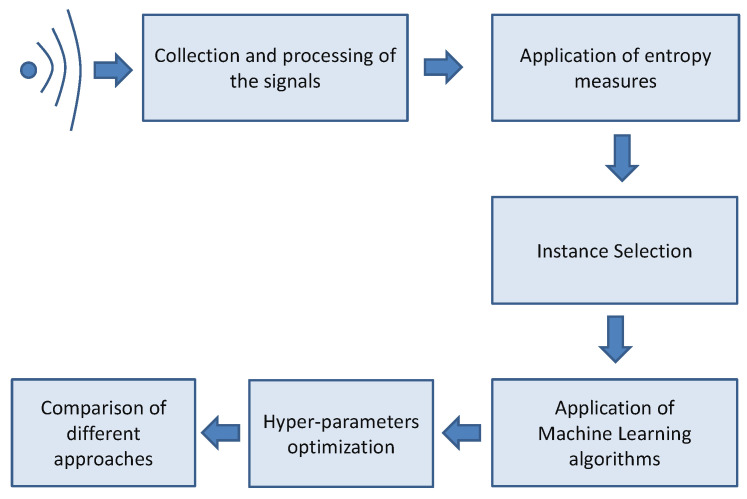
Overall methodology for the spectrum sensing implementation.

The overall methodology for the spectrum sensing implementation is presented in Figure 1. The steps in the methodology are presented below.

In the initial step, the signals of the wireless service are collected, processed, and normalized with mean = 0 and standard deviation = 1 for the application of entropy measures. The signals are then segmented with a fixed segment size which is large enough to include the signal of the wireless service even in the presence of disturbances (fading), which may alter the shape of the signal. The segment size has been calculated to be equal to 110 samples considering that the sampling frequency of the signal is 28 MHz, the radar pulse duration of 1 microsecond, and the distortion of the pulse due to fading effects, which significantly widen the original pulse signal. In addition, the length of the sample is defined to be large enough for the application of the entropy measures while preserving a relatively low computing time. Then, the entropy measures are calculated on the estimated segments (both when the radar pulse is present or absent). The application of the entropy measures generates a feature space on which the ML algorithms are applied. Since the data set (even after the feature reduction due to application of entropy measures) can be quite large and heavily unbalanced, an instance selection method is used to reduce the size of the data set before the application of ML. The instance selection is based on similar approaches in literature where ML is applied to spectrum sensing [28] where a two-step approach is used to reduce the data set, which is given as input to the ML algorithm. Then, a simple sorting algorithm based on the calculation of the feature itself (e.g., ED or FDE) is used to select a subset of the overall feature space to a size $N_{FS}$ < $N_{F}$ where $N_{FS}$ is the size of the obtained subset, and $N_{F}$ is the original size of the data set. The performance of this simple data set reduction approach is evaluated in Section 5, where different data sets of different sizes of $N_{FS}$ are compared. The feature space is divided into a training set and a testing set, where the training set is 2/3 of the overall set and 1/3 is used for the testing phase. In other words, a 3-fold approach is used where the three partitions are created. The process of the creation of the training and testing set is repeated 10 times so that 30 folds are generated to mitigate the risk of over-fitting and to improve generalization of the results. See also Section 3.3.1 for details. The ML processing is applied a number of times to select the optimal values of the hyper-parameters using a grid approach and the choice of the ML algorithm. The probability of detection $P_{d}$ is used as a performance metric for the optimization process. The results of this optimization process are shown in Section 5. Once the optimal values of the hyper-parameters are selected for each entropy measure, the different techniques are compared using the probability detection $P_{d}$ and the probability of false alarms $P_{f}$ (see Section 3.3.2 for the definition of these metrics).

The spectrum sensing scenario chosen to evaluate the proposed approach is related to the coexistence of the weather radars with WLAN/RLAN. This is a well known problem both in the regulatory and research communities in addition to (obviously) the weather radar community [29]. In Europe, most weather radars are operating at C-band (5600–5650 MHz band), sharing the same frequency band with Radio Local Area Network (RLAN) and Wireless Local Area Network (WLAN). Since the World Radiocommunication Conference in 2003 (WRC-03), the primary allocation for Wireless Access Systems including WLAN/RLAN and WLAN was set in the bands of 5.150–5.350 and 5.470–5.725 GHz.

### 3.2. Materials

On the basis of (WRC-03), weather radars and WLAN/RLAN are expected to coexist in the same radio frequency bands due to the conditions defined in ECC/DEC/(04)08 and ETSI in [30]. Then, the WLAN/RLAN is required to implement the Dynamic Frequency Selection (DFS) specified in [30] (i.e., ED) to detect the radar signals and avoid the usage of the corresponding identified radars channels by WLAN/RLAN. Then, one of the objectives of the study presented in this paper is to evaluate alternative implementations of the DFS in addition to the ED. Note that this is a research study, and it does not imply in any way that the authors propose this solution (at the moment of writing this paper) to regulatory and standardization bodies.

The proposed approach is applied to the specific problem of spectrum sensing for weather radar signals because the study presented in this paper was part of a project on radar coexistence with WLAN/RLAN. The proposed approach could be generalized to other types of signals where an incumbent wireless service or PU user must be detected by a secondary user or a wireless services which must coexist in the same frequency band. The FDE is based on the fluctuations of the elements of the time series and the optimal performance is obtained when the FDE (and its variants IFDE and AIFDE) is applied in the time domain. Then, the proposed approach can be adapted to most of the signals of the spectrum sensing problems because the approach will detect the sudden change in the time series (i.e., the digitized signal in space collected by the radio frequency receiver) when the signal to be sensed appears above the noise floor. For the pulse radar signals considered in this study, the proposed approach in this paper is particularly suitable because of the sharp fluctuations in the time domain, but it may have the worst spectrum sensing performance when the signal is not pulse like (e.g., a ramp up signal).

In addition, another potential disadvantage of the approach proposed in this paper is related to the additional computations which must be performed in comparison to simple methods like the ED: the application of FDE with O(N) computing complexity as reported in [8], the averaging step in IFDE, the selection of the optimal hyper-parameters in FDE, IFDE and/or the estimate of the standard deviation for AIFDE. Then, the proposed approach in this study is less suitable when the signal to be sensed is significantly long (i.e., large N).

**Figure 2:**
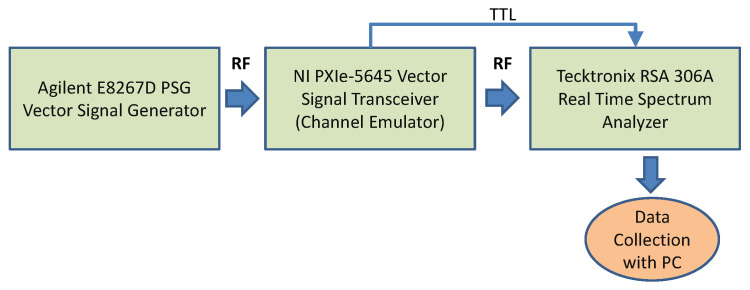
Test bed for the generation of the signals and data collection.

The test bed for the generation of the radar signals is based on the radar test signal defined in [30] on which the evaluation of the proposed approach is performed as described in Figure 2. This is a conducted test bed connected with RF cables. The entire set-up was properly calibrated and the loss of the RF equipment (e.g., RF cables, adapters) was recorded and considered in the measurement and data collection phases. The test bed is composed of the following elements:

- Agilent E8267D PSG Vector Signal Generator: this signal generator was used to create a simulated radar signal with the following settings which are based on the radar test signal defined in [30] sampling frequency: 40.00 MHz, pulse width: 1 microsecond, the first Pulse Repetition Frequency (PRF1): 800 Hz, the second PRF (PRF2) is 1200 Hz. The number of pulses for each PRF is 18. This configuration creates a sequence of pulses with one pulse lasting 1 microsecond, one pause lasting 1.25 ms, one pulse lasting 1 microsecond, and one pause lasting 0.833 ms. A total of 1920 radar pulses were generated plus the pauses. The carrier frequency for the train of radar pulses is set to 5650 MHz in the PSG Vector Signal Generator since this is the frequency where most of the interferences take place in Europe [29].
- The RF channel emulator based on the NI-VST (Vector Signal Transceiver by National Instruments) PXIe-5645R which was extended with additional fading models and configurations. The channel emulator implements the Tapped Delay Line (TDL) model based on the standard 3GPP TR 38.901 version 14.0.0 Release 14 standard (page 66 to 70) [31].
- Tektronix RSA 306A Real-Time Spectrum Analyzer with 40 MHz of bandwidth, which is used to collect the signal output from the RF channel emulator. A sampling frequency of 28 MHz is used to be well within the limits of the Real-Time Spectrum Analyzer.

The digital output from the Real-Time Spectrum Analyzer was collected and recorded in a Personal Computer (PC) for further processing using MATLAB. The recorded digital output is in the time domain in In-phase and Quadrature (IQ) components. Since some entropy measures (e.g., Shannon entropy and Renyi entropy) are applied in the frequency domain, the IQ data in the time domain are also transformed to the frequency domain using Fast Fourier Tranform (FFT). Only the magnitude component of the frequency domain representation was used for detection since the use of the phase component resulted in a degraded performance.

The channel emulator implements five different TDL models which are defined in detail in [31]: three models (TDL-A, TDL-B, and TDL-C) for Non Line of Sight (NLOS) scenarios and two models (TDL-D and TDL-E) for Line of Sight (LOS) scenarios. The models include both Rician and Rayleigh fading conditions.

**Figure 3:**
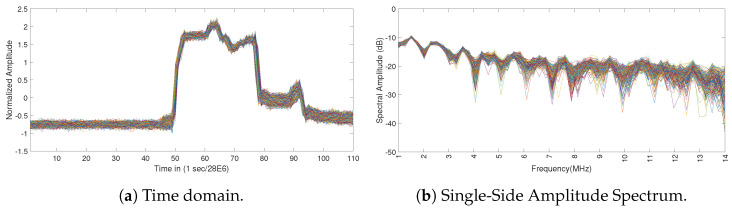
Image of the Weather Radar bursts in fading conditions based on the TDL-A model of [31].

Figure 3 shows the impact of the TDL-A model on the radar pulse. Figure 3a shows the representation of the radar pulse in the time domain. Figure 3b shows the representation of the radar pulse in the frequency domain. It can be seen that the fading condition introduces a distortion in the shape of the pulse, which can negatively impact the detection of the pulse in the implementation of the spectrum sensing function. The presence of noise (e.g., Additive White Gaussian Noise (AWGN)) can further negatively impact the detection performance as shown in Section 5.

### 3.3. Machine Learning Algorithms and Classification Metrics

#### 3.3.1. Machine Learning Algorithms

The following machine learning algorithms have been used to evaluate the performance of the detection process.

- SVM is a supervised learning model with the related learning algorithms that classify data by creating a hyperplane or set of hyperplanes in a high- or infinite-dimensional space, to distinguish the samples belonging to different classes. Various kernels have been tried and the one providing the best performance was the Radial Basis Function (RBF) kernel, where the values of the scaling factor γ must be optimized together with the parameter *C* [32].
- KNN is an approach to data classification that estimates how likely a data point is to be a member of one class or another depending on what group the data points nearest to it are in. The KNN is an example of a lazy learner algorithm, meaning that it does not build a model using the training set until a query of the data set is performed. The main hyperparameter in KNN is the *K* factor, which must be optimized for the specific classification problem. The type of distance metric used to calculate the ’nearest’ must also be chosen carefully.
- Decision tree is a predictive modeling approaches where a decision tree (as a predictive model) analyzes the observations about an item (represented in the branches) to reach conclusions about the item’s target value (represented in the leaves). In this case, we use classification trees where leaves represent class labels and branches represent conjunctions of features that lead to those class labels. The hyper-parameter chosen for optimization is the maximum number of branches $N_{B}$ at each split. The option in which the algorithm trains the classification tree learners without pruning them was chosen.

#### 3.3.2. Detection Metrics

The classification metrics adopted on this paper are adopted on the basis of two considerations. The first consideration is that they are commonly used in literature to evaluate the performance of the spectrum sensing algorithm [4,6,15]. The second consideration is they are derived from the binary hypothesis test, which is used to find out the presence of the PU or incumbent signal: $H_{0}$ is the noise in the absence of a PU/incumbent signal and $H_{1}$ indicates the presence of a PU/incumbent signal as defined in the following equations:

(1) $$H_{0}\to x_{i}=u\left(i\right),i=1,\ldots ,N$$

(2) $$H_{1}\to x_{i}=s\left(i\right)+u\left(i\right),i=1,\ldots ,N$$

where s(i) is the PU/incumbent signal, u(i) is the noise and *i* = 0, 1, 2, *…*, *N*, is the sample size under analysis. Then, $H_{0}$ indicates the absence of a PU/incumbent signal, and $H_{1}$ indicates the presence of a PU/incumbent signal.

Then, the following evaluation metrics are defined:

- The detection probability $P_{d}$ refers to the numbers of correct detections (PU is present) over the total number of sensing operations. Another definition of $P_{d}$ is the probability of deciding $H_{1}$ when $H_{1}$ is true.
- The probability of false alarm $P_{f}$ refers to the number of times that the PU is falsely detected over the total number of sensing operations. Another definition of $P_{f}$ is the probability that the decision is $H_{1}$ when $H_{0}$ is true.

## 4. Entropy Measures

In the rest of this section, $X=x_{i},x_{i+1},\ldots ,x_{N}$ is the time series under analysis, which represents the window of the data of the signal collected in the spectrum sensing process (e.g., in our study, the window of data are set to a length of 110 samples). The Shannon entropy and Renyi entropy are also applied to the spectral domain representation *XF* of *X* using the Discrete Fourier transform (DFT), which is represented with the following equations:

(3) $$XF\left(k\right)=\sum_{i=1}^{N}x\left(i\right)W_{n}^{\left(i-1)(k-1\right)}$$

where

(4) $$W_{N}=e^{(-2\pi j)/N}$$

where $W_{N}$ is one of the *N* roots of unit.

### 4.1. Shannon Entropy

The Shannon entropy is defined as follows:

(5) $$SE=-\sum_{i}^{N}p\left(x_{i}\right)log\left(p\left(x_{i}\right)\right)$$

where $p(x_{i})$ is the probability $p(x=x_{i})$.

Note that, in this paper, the Shannon entropy measure is applied to the spectral domain representation (amplitude only) |XF| of the signal *X* as it provides a superior performance in spectrum sensing [4].

### 4.2. Renyi Entropy

The Renyi entropy of order *o* is defined in the following Equation (6):

(6) $$RE=\frac{1}{1-o}log\left(\sum_{i}^{N}p{\left(x_{i}\right)}^{o}\right)$$

where $p(x_{i})$ is the probability $p(x=x_{i})$. The limit for o⟶1 is the Shannon Entropy defined above. In this paper, we adopt the values of o=2,3,4 as this is the range of values used in literature [6].

As in the case of Shannon entropy, the Renyi entropy measure is applied to the spectral domain representation (amplitude only) |XF| of the signal *X* because it provides a superior performance in spectrum sensing [6].

### 4.3. Dispersion Entropy

DE was recently introduced in [7] and refined in [8], and it addresses the potential weakness of Permutation Entropy (PE) where the mean value of amplitudes and differences between amplitude values are not considered in its definition. In DE, the initial series $X=x_{i},x_{i+1},\ldots ,x_{N}$ (the window of the data of the signal collected in the spectrum sensing process) is mapped to *c* classes. While this mapping can be implemented with various linear or nonlinear approaches, the authors in [7] propose to use Normal Cumulative Distribution Function (NCDF) to map *X* to the *c* classes. This mapping function (called $M_{F}$ in the rest of this paper) has been extended in [8] to other functions. Then, the initial time series *X* is transformed to another time series *U* with $U=u_{i},u_{i+1},\ldots ,u_{N}$. To make a comparison with the performance of DE in the spectral domain, the DE can also be applied to XF presented in Section 4. In both cases of DE and FDE and the subsequent improved variants IDE and IFDE introduced in this study, the mapping between the original signal *X* ($x_{i}$) and *U ($u_{i}$)* can be implemented in different ways: using a simple linear function or more sophisticated functions like the sigmoid, the hyperbolic tangent sigmoid function, the normal continuous distribution function and so on. Depending on the structure of the time series, the choice of the mapping function can produce better or worst results for the spectrum sensing detection. Then, the choice of the mapping function becomes another hyper-parameter to be optimized in the implementation of DE and FDE together with *m* and *c* (the delay parameter is set to 1 for the reasons described before, which are to avoid the risk of bypassing relevant samples for spectrum sensing). In this paper, we use the same mapping functions $M_{F}$ described in [8].

The following terminology for the mapping functions is used in the rest of this paper:

- ‘LM’ (linear mapping);
- ‘NCDF’ (normal cumulative distribution function);
- ‘TANSIG’ (tangent sigmoid);
- ‘LOGSIG’ (logarithm sigmoid);
- ‘SORT’ (sorting method).

An additional transformation is performed on *U* where dispersion patterns are generated from sequences of *U* with embedding dimension *m* and delay *d* as in the following formula (note that it is adopted a similar notation to [8]):

(7) $$u_{i}^{m,c}=u_{i}^{c},u_{i+d}^{c},\ldots ,u_{i+(m-1)d}^{c},i=1,2,\ldots ,N-\left(m-1\right)d.$$

Then, each time series $u_{i}^{m,c}$ is mapped to a dispersion pattern $\pi_{v0v1\ldots v(m-1)}$, where $u_{i}^{c}=v0,u_{i+d}^{c}=v1,\ldots ,u_{i+(m-1)d}=v_{m}-1$. The number of possible dispersion patterns is equal to $c_{m}$, since $u_{i}^{m,c}$ has *m* elements, and each can be one of the integers from 1 to *c*. This equality does also impose a condition on the values of the hyper-parameters *c*, *m*, and *d* used in the analysis since $c^{m}<=N$ with d=1 as in the case of this study because no delay is used to avoid the risk of missing relevant signal samples indicating the presence of the signal in space to be sensed.

For each $c_{m}$ dispersion patterns $\pi_{v0v1\ldots v(m-1)}$, the relative frequency is obtained as follows:

(8) $$p\left(\pi_{v0v1\ldots v(m-1)}\right)=\frac{\#\left\{i|i<=N-\left(m-1\right)d,u_{i}^{m,c}\in \pi_{v0v1\ldots v(m-1)}\right\}}{N-(m-1)d}$$

where # has the meaning of cardinality and ∈ has the meaning ’has type’ in this context. Then, by applying the Shannon definition of entropy to the dispersion patterns, the final value of the DE is calculated with the following equation:

(9) $$DE\left(x,m,c,d\right)=-\sum_{\pi =1}^{c^{m}}p\left(\pi_{v0v1\ldots v(m-1)}\right)\cdot log\left(p\left(\pi_{v0v1\ldots v(m-1)}\right)\right)$$

The MATLAB implementation of DE provided by the authors of [7] was used in this paper.

### 4.4. Fluctuation Dispersion Entropy

A variation of the DE was introduced in [8] where only the differences between adjacent elements of dispersion patterns are considered. Such fluctuations are termed fluctuation-based dispersion patterns. To make a comparison with the performance of FDE in the spectral domain, the DE can also be applied to *XF* presented in Section 4 in addition to *X*.

In this case, the patterns have dimension (m−1) where each of their elements changes from (−c+1) to (c−1). Then, the number of fluctuation-based dispersion patterns becomes ${\left(2c-1\right)}^{\left(m-1\right)}$, which imposes a limit on the values of the *c* and *m* parameters because ${\left(2c-1\right)}^{\left(m-1\right)}<N$. This variation of DE is called FDE in the rest of this paper. In FDE, the dispersion pattern 1, 3, 4 is equivalent to 2, 4, 5 and in a similar way 1, 1, 1 is equivalent to 3, 3, 3. One example on the application of FDE is provided here. If there is a signal *x* = 3, 4.5, 6.2, 5.1, 3.2, 1.2, 3.5, 5.6, 4.9, 8.4 and we have *m* = 3, *c* = 2 and *d* = 1, we have $3^{2}$ potential fluctuation dispersion patterns ((−1, −1), (−1, 0), (−1, 1), (0, −1), (0, 0), (0, 1), (1, −1), (1, 0), (1, 1)). With two classes and related values 1, 2 the original signal *x* is transformed to (1, 1, 2, 2, 1, 1, 1, 2, 2, 2). Then, a sliding window of size *m* = 3 is used to estimate the differences between adjacent elements of dispersion patterns, which produces ((0, 1), (1, 0), (0, −1), (−1, 0), (0, 0), (0, 1), (1, 0), (0, 0)). Finally, the number of each fluctuation-based dispersion pattern is counted.

The rationale for the application of FDE to the problem of spectrum sensing is that the detection of the PU signal in space is more related to the fluctuations of the signal rather than the distribution of the dispersion patterns as in other domains (e.g., specific patterns in an electrocardiogram). Then, FDE can be well adapted to this problem of spectrum sensing. This initial assumption will be validated by the results presented in Section 5.

### 4.5. Improved Fluctuation Dispersion Entropy

The fluctuations in the time series on which the FDE is implemented may be sensible to the presence of noise. In this specific context of spectrum sensing, an improvement of the FDE is proposed where the *U* series is created not directly from *X* but from another representation *Y* where ${\bar{y}}_{i}=\sum_{k=i-S_{FDE}}^{k=i+S_{FDE}}\left(x_{k}\right)$ and the overline symbol $\bar{y}$ represents the mean. In plain words, *U* is created from *Y*, which is in turn created as an average of *X* on a segment of size $S_{FDE}$. This additional operation, which can (and is) be easily integrated in the DE and FDE definition, basically implements a simple smoothing filter, which can improve the robustness of FDE against the noise, and it is particularly suited to the specific problem of spectrum sensing where the noise may significantly degrade the performance of the detector. For an analysis of smoothing filters using averaging, the reader can refer to [33]. The potential trade-off is that this operation may remove the discriminating features which are used for spectrum sensing in this context or for classification in other domains where FDE is applied. This trade-off will be discussed more in detail in Section 5. Then, $S_{FDE}$ becomes another hyper-parameter in the application of FDE to spectrum sensing with a range, which should be significantly less than the length of the signal to be detected (i.e., the radar pulse in this case) but also greater than *m*. This the reason why the approach proposed in this paper is not fully “blind spectrum sensing” because the range of $S_{FDE}$ is between *m* and smaller than the size of the pulse in ideal conditions (which is 28 samples for a radar pulse of 1 microsecond sampled at 28 MHz). Note that this technique can be applied both to FDE but also to DE. The derivations of FDE and DE applying this technique are called respectively Improved FDE (IFDE) and Improved DE (IDE) in the rest of this paper.

To overcome the presence of another hyperparameter $S_{FDE}$ (beyond *m* and *c*) which should also be tuned, an adaptive technique to select the optimal value of $S_{FDE}$ is presented in this study. The technique is based on the consideration that the presence of noise in spectrum sensing changes the standard deviation of the signal in space. Then, the proposed technique calculates the value of $S_{FDE}$ on the basis of the standard deviation of the signal itself. This is a simple calculation, which is not computing intensive. In the adaptive technique, $S_{FDE}$ is directly correlated with the standard deviation, and it is matched to the range of the standard deviation calculated on the range described before (between *m* and the length of the PU signal). In a practical application, it can be calculated in a training phase or directly on a large number of received signals. The application of this adaptive technique to IFDE and Improved Dispersion Entropy (IDE) generates other two variations of FDE and DE called in this paper AIFDE and Improved Dispersion Entropy (AIDE).

The results shown in Section 5 demonstrate that the technique is quite effective: even if it does not provide the exact optimal value of $S_{FDE}$ in all fading scenarios or noisy scenarios, it identifies an optimal value of $S_{FDE}$ which provides a detection performance better than the large majority of the fixed values of $S_{FDE}$ and effectively removes (if the technique is adopted) the need to estimate one hyper-parameter (i.e., the value of $S_{FDE}$).

Note that IDE, IFDE, AIDE, and AIFDE are only applied to the time domain and the related definition on the spectral domain is not provided here for this reason.

## 5. Results

### 5.1. Hyper-Parameter Optimization

The aim of this section is to evaluate the impact of the hyper-parameters defined in the previous sections of this paper (e.g, *m*, *c* $S_{FDE}$) on the detection performance of the spectrum sensing function.

As described before, the optimal values were identified using a grid approach across the folds and the maximum occurrence of the hyper-parameters values in the resulting histogram. While the number of hyper-parameters may seem large, there are some constraints on the range allowed for the hyper-parameters. The hyper-parameters *m* and *c* are constrained by the size of the window of analysis in spectrum sensing: in DE $c^{m}<N$ while in FDE and IFDE ${\left(2c-1\right)}^{\left(m-1\right)}<N$. For the mapping function $M_{F}$, five different functions are evaluated. The value of $S_{FDE}$ can be effectively replaced with the adaptive technique in most scenarios.

Then, this section is focused on the evaluation of the impact of the hyper-parameters described in Table 1 with a grid approach. The results of the evaluation are presented in the form of graphs and figures where the impact of one hyper-parameter or the set of hyper-parameters is shown while the values of the other hyper-parameters are kept constant.

Because the optimization process can be quite time-consuming, it is performed on a reduced size of the initial data set using the instance selection process described in Section 3. The selected data set was reduced to 0.02 of the initial data set. This size was chosen because it still contains a relevant number of samples (around 10,000 samples), but it also has a degree of unbalance between the labels indicating the presence of the pulse radar or the absence of it (pulse radars were present roughly on a ratio of 1 to 5 in the reduced data set). The evaluation of the impact of the different sizes of the data set is shown later in this section. The optimization results are obtained using the Decision Tree (DT) algorithm. An evaluation of the performance of the different ML algorithms is provided later in this section.

Table 1 provides a summary on the identified hyper-parameters and the range of values on which the optimization was performed.

**Figure 4:**
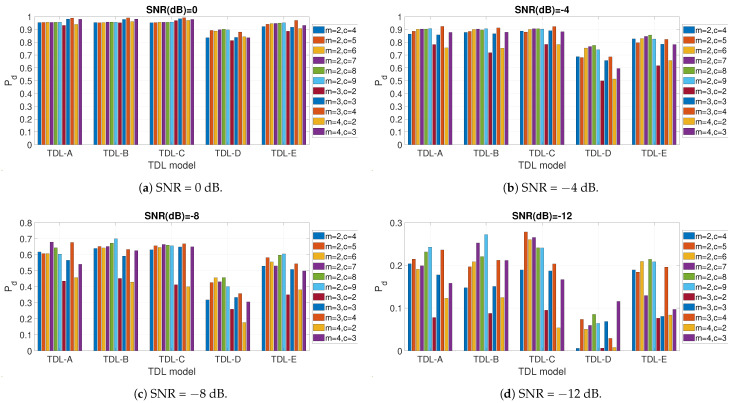
Impact of *m* and *c* values for the different fading models and different values of SNR in dB.

The first result presented in this section is the impact of the values of the *m* and *c* hyper-parameters, which is shown in Figure 4 and related subfigures for different fading models (e.g., TDL-B) and different values of SNR in dB. Because of the large quantity of results to present, only the values of SNR = −12, −8, −4.0 dB are shown. As demonstrated in subsequent figures, these values of SNR are the most relevant for the evaluation of the impact of the hyper-parameters. The graphs are obtained using the FDE.

The results show that the impact of the choice of *m* and *c* is relevant, and it may vary across different fading conditions and values of SNR. One common trend, which can be extracted from the presented results, is that a higher value of *c* (the number of classes) usually supports a higher detection accuracy (e.g., a higher value of $P_{d}$). On the other side, we remind that the value of *c* is bound to an upper limit as described before, and such limits cannot be increased further. Similar considerations can be proposed for *m*. Then, an evaluation of the results shows that the combination of m=3 and c=4 provides in most cases the optimal detection accuracy measured with $P_{d}$, in particular for higher values of SNR expressed in dB. For this reason, the values of m=3 and c=4 will be used in the rest of this paper. A potential reason why these values are optimal is that a relative large number of classes (c=4) supports a better classification between the cases of presence or absence of signal.

**Figure 5:**
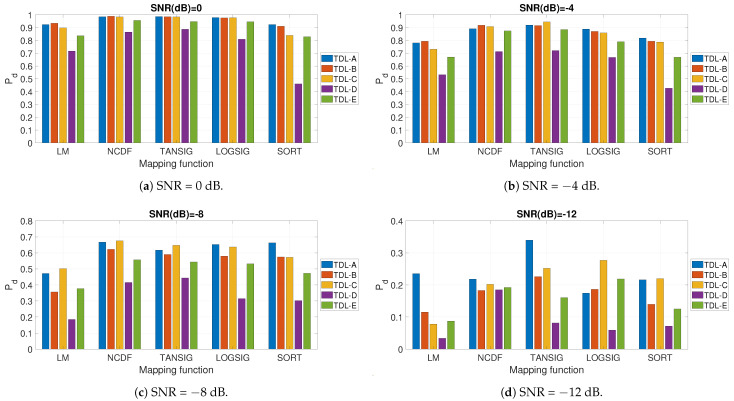
Impact of the mapping functions for different fading models and different values of SNR in dB.

Then, the impact of the mapping functions between the initial time series *X* (or $\left|XF\right|$ if the frequency domain amplitude is used) and *U* were evaluated in a similar way to the previous results. The results are shown in Figure 5 and related sub-figures, and they are obtained using the FDE with m=3 and c=4 and the optimal values of the other hyper-parameters shown in Table 1. The results are calculated for different fading models (e.g., TDL-B) and different values of SNR in dB. Due to the large quantity of results to present, only the values of SNR = −12,−8,−4.0 dB are shown. The results show that the best detection performance is obtained with the TANSIG mapping function especially with relatively high values of SNR in dB (i.e., 0 dB and −4 dB) across the different fading models. The NCDF function also obtains a very good performance in particular for lower values of SNR in dB (i.e., −8 dB and −12 dB). The reason why the TANGSIG and NCDF mapping functions obtain a higher $P_{d}$ than the other mapping functions like the linear mapping (LM) or the sorting algorithm (SORT) is related to the consideration that TANSIG is able to capture in a more efficient way the nonlinearities in the signal in the presence of fading effects or noise. In other words, the greater slope of TANSIG and NCDF in comparison to the linear mapping LM means that they show a greater response to a small deviation in the signal in space (i.e., the fluctuations). Therefore, the ML algorithm can better distinguish between small variations in the signal, and it can generate a much more nonlinear response, which supports a more effective classification.

**Figure 6:**
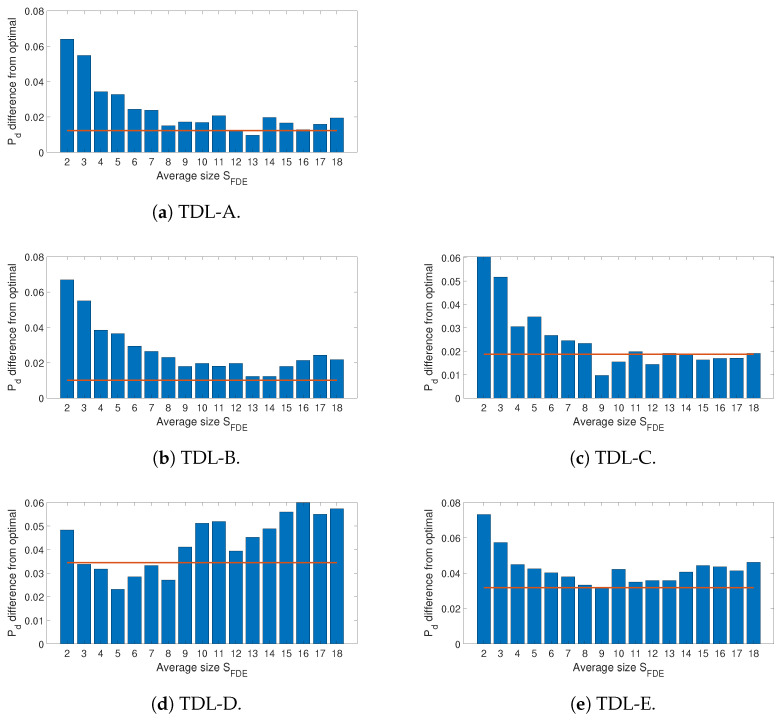
Sum of the differences for $P_{d}$ from the optimal value (across all the values of SNR considered in the study) for different window sizes $S_{FDE}$ using IFDE. The line indicates the sum of the differences for the adaptive method (i.e., AIFDE) to show the comparison with the different values of the window size $S_{FDE}$. Each figure represents a different channel propagation model (i.e., TDL from [31]).

As described in Section 3, the approach proposed in this paper is not only based on the application of DE and FDE but also on their improved versions IDE and IFDE where the transformation from *X* to *U* includes an averaging step (calculated on the basis of the parameter $S_{FDE}$) for the neighbors of the selected sample *i*. In addition, the definitions of IDE and IFDE were further improved with an adaptive step where $S_{FDE}$ is adaptively calculated to obtain the AIDE and AIFDE. Then, an initial evaluation is done to see how AIDE and AIFDE perform in comparison to IDE and IFDE for a range of $S_{FDE}$ values. The next set of figures (Figure 6 and related sub figures) show the impact of the parameter $S_{FDE}$ on the detection performance. The results presented in the figures are generated using IFDE and by averaging the differences between the maximum value of $P_{d}$ obtained at each SNR for an optimal value of $S_{FDE}$ and the $P_{d}$s obtained for a fixed value of $S_{FDE}$ (represented by a specific bar in the bar graph).

In addition, the performance with AIFDE is also presented in Figure 6 and related sub figures using a line over-imposed on the bar graph. It can be seen that the approach based on AIFDE is able to outperform the application of IFDE with fixed values of $S_{FDE}$ in most cases and across the different fading models (in particular for TDL-A, TDL-B, and TDL-E). For the TDL-C and TDL-D fading models, AIFDE is able to outperform IFDE for the majority of the fixed values of $S_{FDE}$ even if $S_{FDE}$ values equal to 8 or 9 provide an excellent detection performance as well. On the other side, the adoption of AIFDE allows for avoiding the task to select the optimal value of the $S_{FDE}$ hyper-parameter.

**Figure 7:**
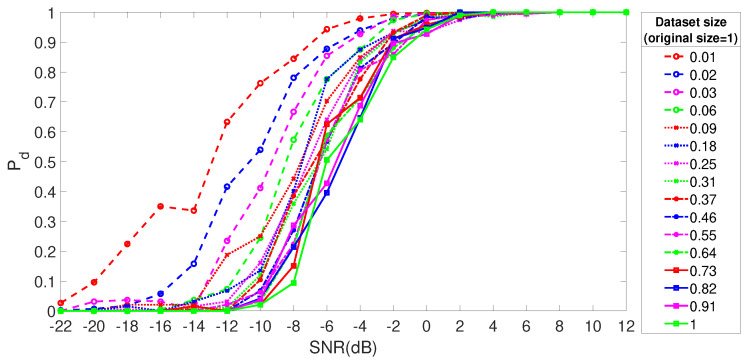
$P_{d}$ with different sizes of the data sets reduced from the initial data set through the instance selection process. AIFDE was used.

**Figure 8:**
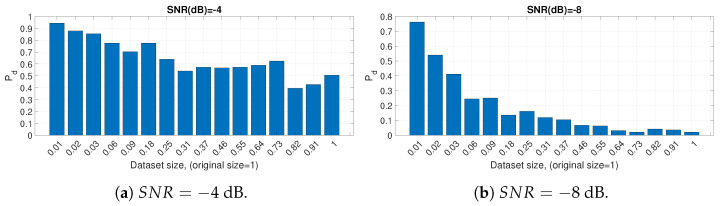
Bar graphs of the $P_{d}$ with different sizes of the data sets reduced from the initial data set through the instance selection process for SNR=−4 dB and SNR=−8 dB. AIFDE was used.

All the results shown so far in this section are based on a data set, which is reduced to a size of 0.02 of the initial data set with the instance selection method described in Section 3. Then, we evaluated the performance of the instance selection algorithm in relation to the baseline with the whole data set using $P_{d}$ as the metric. Ideally, the instance selection algorithm should be able not only to minimize the size of the data set but also to maintain or improve the detection performance. The $P_{d}$ trend in comparison to the SNR for the TDL-A fading model (the other fading models show a similar result and they are not displayed for space reasons) is shown in Figure 7 for AIFDE for different sizes of the data set reduced by the instance selection algorithm. We note that the algorithm is able not only to reduce the data set (and therefore the classification time) but also to improve the detection performance and robustness to noise. The same results shown in Figure 7 are described more in detail and clarity for specific values of SNR in Figure 8a for SNR = −4 dB and Figure 8b SNR = −8, which confirm the previous statements since it can be seen that smaller data sizes enhance the detection performance. The reason why the instance selection algorithm is able to improve the detection performance in this particular case of spectrum sensing is because this simple algorithm mostly excludes samples where the signal is absent. For example, in the case of the ED, the energy of the data window of the signal in space is higher than the energy of the data window when the signal is absent. Then, the machine learning algorithm will operate on a reduced data set where the data model has a higher population ratio of samples where the signal is present than when it is not present, and the detection of the signal will be more effective. The trade-off of the application of this pre-processing step is that it requires additional computing resources.

We note that this approach is not new in the context of spectrum sensing because a similar pre-processing step has already been adopted in literature with similar results [17,28,34].

The performance of IFDE and IDE was compared with the application of the basic definition of FDE and DE both in the time domain and the spectral domain to evaluate in a quantitative way the improvement in detection performance of IFDE and IDE.

**Figure 9:**
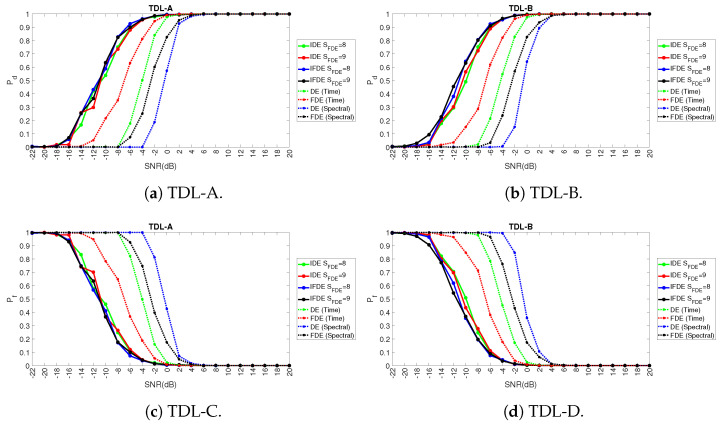
Results for the comparison of IFDE and IDE against FDE and DE on the basis of $P_{d}$ and $P_{f}$ across different TDL models and different levels of SNR.

Figure 9 and related sub figures show the $P_{d}$ and $P_{f}$ for different values of SNR in dB and the fading models TLD-A and TDL-B (for reasons of space, the results of the other fading models are not presented, but they provide similar results). It can be seen that the IFDE and IDE for selected values (here it is represented only the IFDE and IDE in the time domain) of $S_{FDE}$ significantly outperform FDE and DE both in the time domain and spectral domain. In particular, it can be noticed that the detection performance of FDE and DE in the time domain is better than the frequency domain (while the opposite is true in the case of SE and RE). A potential reason for this result is that the dispersion patterns related to the presence of the signal in space (in comparison to the absence of signal) are more visible in the time domain even in the presence of Gaussian noise rather than in the spectral domain. For this reason, in the rest of this paper, the IFDE and IDE (and its adaptive variants AIFDE and AIDE) are only calculated in the time domain.

The results in Figure 9a,b (for $P_{d}$) and Figure 9c,d (for $P_{f}$) also show that the performance of FDE is slightly better than its counterparts DE (and similarly for IFDE and IDE). This is particularly visible for FDE and DE in both fading models TDL-A and TDL-B in Figure 9a,b, and it is slightly visible for IFDE and IDE in particular for the TDL-B model in Figure 9b. This is due to the reason that the FDE and IFDE are focused on the analysis of the fluctuations (i.e., differences) between subsequent samples which is more effective in the detection of the presence of the PU signal over the noise floor in comparison to the analysis of the dispersion patterns from DE and IDE.

**Figure 10:**
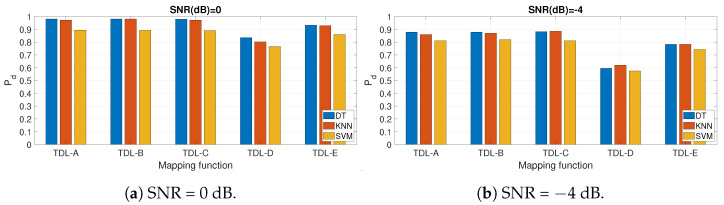
$P_{d}$ for different machine learning algorithms. AIFDE is used.

Finally, we perform a comparison of the different ML algorithms identified in Section 3. The results for different values of SNR and for the different fading models are shown in Figure 10. It can be seen that DT outperforms the other ML algorithms like KNN and SVM. This is why DT is used to generate the results presented in this section.

This last result completes the evaluation of the impact of the hyper-parameters for the detection performance in the spectrum sensing function. The next sub-section compares the detection performance of the proposed approach with the approaches proposed in literature based on the ED and other entropy measures.

### 5.2. Comparison with Other Approaches from Literature

**Figure 11:**
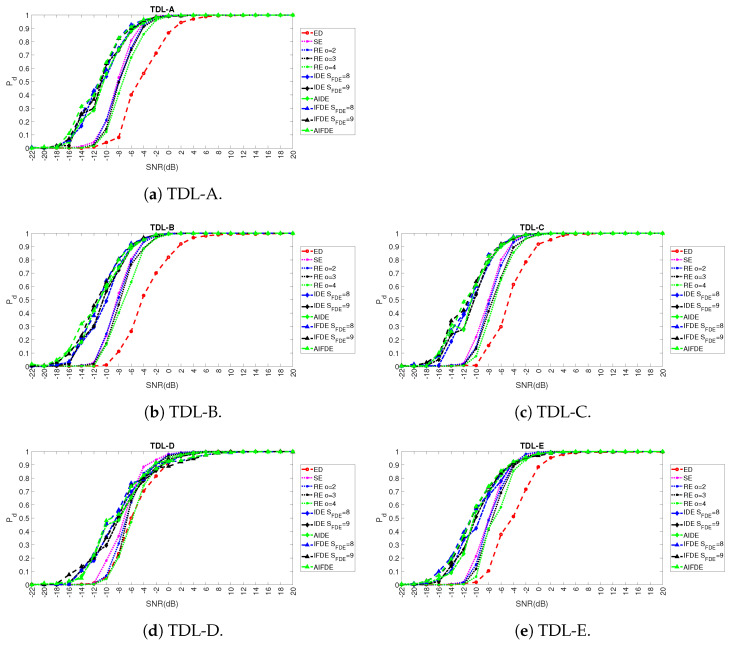
Results for the comparison of $P_{D}$ across different TDL models.

**Figure 12:**
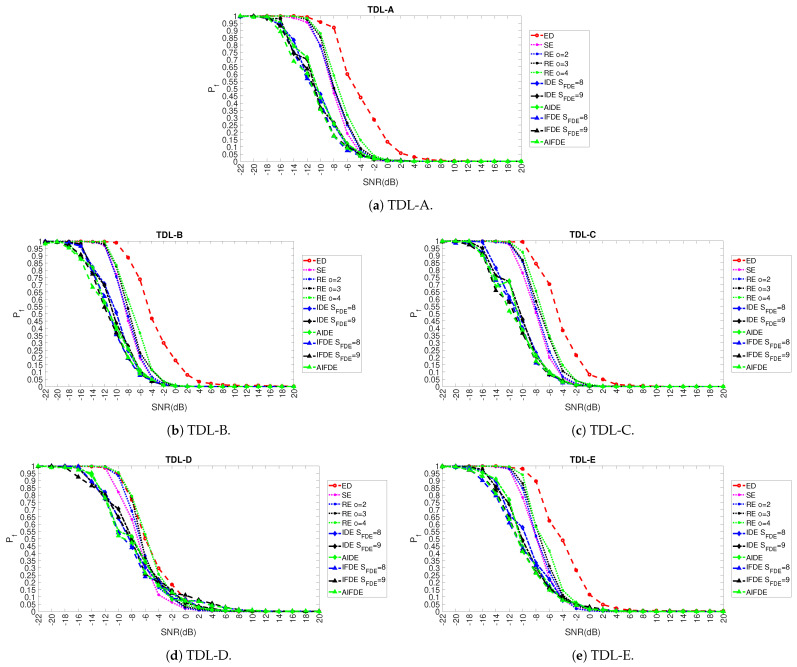
Results for the comparison of $P_{f}$ across different TDL models.

Once the values of the optimal hyper-parameters (at least in the majority of the fading scenarios) have been selected as described in the previous section, a comparison with the methods used in literature is performed. In particular, we compare the results obtained with AIFDE, AIDE, IFDE, IDE (for two different values of the window $S_{FDE}$) with the ED (which is the baseline detector used in spectrum sensing) and the other entropy measures used in literature for spectrum sensing: Shannon entropy [4,16] and Renyi Entropy of order *o* = 2, 3 and 4 [6]. The results are shown in Figure 11 (for $P_{d}$) and Figure 12 (for $P_{f}$) and related subfigures for different values of SNR in dB and for the five adopted fading models: TDL-A, TDL-B, TDL-C, TDL-D, TDL-E.

It can be seen that AIFDE, AIDE, IFDE, IDE are generally able to outperform significantly the baseline ED, Shannon entropy, and Renyi entropy detectors in a coherent manner across the five adopted fading models, and they are generally more robust in the presence of noise. All the entropy measures provide better results against the ED across all the fading models and the different levels of SNR in dB. In general, the AIFDE and IFDE (blue and green line with triangular markers) are able to obtain the optimal detection performance over the approaches based on the other entropy measures with a significant gain in dB (e.g., 3 dB for AIFDE in comparison to SE and 8 dB for AIFDE in comparison to ED for $P_{D}=0.7$). Only in the case of the TDL-D fading model, it can be seen from Figure 11d and Figure 12d that SE and RE outperform AIFDE, AIDE, IFDE, and IDE for high values of SNR (i.e., SNR > −6 dB) even if AIFDE, AIDE, IFDE, and IDE are more robust in the presence of noise. The potential reason for this behavior is that the averaging step introduced in IDE, IFDE, AIDE, and AIFDE can improve the robustness of the algorithm in the presence of noise, but it can decrease the contribution of the discriminating features in the detection process for this specific type of fading model. Still, AIFDE, AIDE, IFDE, and IDE are significantly better than the ED even in the TDL-D fading model. As described before, the reason for such improvement is that the FDE and its variants IFDE and AIFDE focus on the fluctuations of the time series, which are more evident in the spectrum sensing problem, where the objective is to detect the presence of the signal in space in comparison to the case where the signal is absent. To clarify better the performance of the approach proposed in this paper with the different approaches proposed in literature, Table 2 shows the specific values of $P_{d}$ and $P_{f}$ obtained for the values of SNR =−12 dB and SNR =−8 dB for the five different fading models TDL-A, TDL-B, TDL-C, TDL-D, and TDL-E.

The better performance of the approaches based on the IDE, IFDE, AIDE, and AIFDE measures has the price of increasing the computing time needed to calculate the related entropy measures. An estimate of the computing time necessary to generate the feature matrix for the subsequent application of ML shows that the application of AIFDE and AIDE is roughly four times the time needed by the ED, the application of IFDE and IDE is roughly 3.5 times needed by the ED, and the application of Shannon entropy and Renyi entropy are roughly 1.2 the times needed by the ED.

## 6. Conclusions

This paper proposed the recently introduced Dispersion entropy and Fluctuation Dispersion entropy measures to the problem of spectrum sensing. In particular, a new improved definition of Dispersion entropy and Fluctuation Dispersion entropy is applied to enhance the robustness against noise, which are called respectively IDE and IFDE. The improvement is based on the introduction of an averaging step introduced in the data conversion. Because the IDE and IFDE introduce a new hyper-parameter, the study presented in this paper proposes an adaptive variation of IDE and IFDE (respectively called AIDE and AIFDE), where the value of the hyper-parameter is automatically estimated from the standard deviation of the signal. This variation of DE and FDE is particularly suited to the problem of spectrum sensing where the presence of noise or disturbance like fading can negatively impact the classification performance. The approach is applied to radar signals generated in the laboratory with a hardware platform implementing the channel emulator to generate different fading conditions. The results of the proposed approach are compared with the ED and other entropy measures commonly used in literature for spectrum sensing: Shannon entropy and Renyi entropy. The results show that IDE, IFDE, AIDE, and AIFDE are able to outperform (in terms of detection probability $P_{d}$ and false alarm probability $P_{f}$) the ED with a large margin and the Shannon entropy and Renyi entropy with a significant margin across the five different fading conditions and for different conditions of SNR. The price for the improved performance of IDE, IFDE, AIDE, and AIFDE is a slightly higher computing time in the implementation of the spectrum sensing function.

The approach proposed in this paper is particularly suitable for the implementation of spectrum sensing for pulse like signals because the FDE, IFDE, and AIFDE will exploit the fluctuations of the signal in space against the noise floor. It may be less suitable for signals with non-pulse shape (e.g., a ramp shaped signal) because the fluctuations will be less evident. In addition, the additional computations needed to implement the spectrum sensing approach (estimate of FDE, averaging step in IFDE or estimate of the optimal hyper-parameters) may generate a negative performance impact with signals of long duration. Future developments will analyze the proposed approach with these latter types of signals and investigate methods to mitigate these weaknesses.

## Figures and Tables

**Table 1:** Identification and description of the main hyper-parameters used in this study including their optimal values and the range where the optimization was performed.

Hyper-Parameters of the Machine Learning Algorithms	Parameters
SVM	RBF scaling factor $\gamma =2^{5}$ (range: $2^{1}$ to $2^{10}$), *C* factor = $2^{8}$ (range: $2^{1}$ to $2^{10}$)
KNN	K=1 (range: 1 to 10), Distance metric = Euclidean Distance (choices: Chebychev, Euclidean, Minkowski)
Decision Tree	$N_{B}=8$ (range: 2 to 12), Split Criterion = Gini’s diversity index (choices: Gini Index and Cross-Entropy)
**Hyper-parameters of the entropy measures**	
DE	embedding dimension m=3 (range 2, 3, 4), number of classes c=4 (range 2, 3, …, 9). Mapping function equal to TANSIG with range (LM, NCDF, TANSIG, LOGSIG, SORT).
IDE and AIDE	embedding dimension m=3 (range 2, 3, 4), number of classes c=4 (range 2, 3, …, 9), averaging parameter $S_{FDE}=8$ or adaptive calculation of $S_{FDE}$. Mapping function equal to TANSIG with range (LM, NCDF, TANSIG, LOGSIG, SORT).
FDE	embedding dimension m=3 (range 2, 3, 4), number of classes c=4 (range 2, 3, …, 9). Mapping function equal to TANSIG with range (LM, NCDF, TANSIG, LOGSIG, SORT).
IFDE and AIFDE	embedding dimension m=3 (range 2, 3, 4), number of classes c=4 (range 2, 3, …, 9), averaging parameter $S_{FDE}=9$ or adaptive calculation of $S_{FDE}$. Mapping function equal to TANSIG with range (LM, NCDF, TANSIG, LOGSIG, SORT).

**Table 2:** Comparison of the approaches: detailed values of $P_{d}$ and $P_{f}$ for SNR =−12 dB and SNR =−8 dB.

Approach	$P_{d}$ (SNR = −12 dB)	$P_{d}$ (SNR = −8 dB)	$P_{f}$ (SNR = −12 dB)	$P_{f}$ (SNR = −8 dB)
TDL-A				
ED	0.0083	0.08	0.9916	0.919
Shannon entropy	0.00447	0.5322	0.955	0.4677
Renyi entropy o=2	0.0255	0.5162	0.975	0.50
Renyi entropy o=3	0.028	0.4953	0.975	0.504
Renyi entropy o=4	0.0156	0.4104	0.984	0.589
IDE ($S_{FDE}=8$)	0.4229	0.7515	0.577	0.248
IDE ($S_{FDE}=9$)	0.2984	0.735	0.714	0.264
AIDE	0.2854	0.742	0.701	0.2578
IFDE ($S_{FDE}=8$)	0.4322	0.826	0.567	0.1694
IFDE ($S_{FDE}=9$)	0.365	0.823	0.634	0.1706
AIFDE	0.3958	0.827	0.604	0.1729
TDL-B				
ED	0.001	0.1125	0.9989	0.8875
Shannon entropy	0.0029	0.5489	0.973	0.451
Renyi entropy o=2	0.021	0.516	0.98	0.4833
Renyi entropy o=3	0.019	0.464	0.98	0.535
Renyi entropy o=4	0.012	0.401	0.998	0.598
IDE ($S_{FDE}=8$)	0.294	0.752	0.705	0.247
IDE ($S_{FDE}=9$)	0.302	0.723	0.6975	0.276
AIDE	0.42	0.741	0.575	0.258
IFDE ($S_{FDE}=8$)	0.38	0.801	0.619	0.1937
IFDE ($S_{FDE}=9$)	0.455	0.8	0.577	0.1963
AIFDE	0.425	0.806	0.585	0.187
TDL-C				
ED	0.0078	0.1567	0.992	0.843
Shannon entropy	0.0023	0.4953	0.976	0.5047
Renyi entropy o=2	0.0177	0.467	0.982	0.5328
Renyi entropy o=3	0.0093	0.414	0.986	0.5854
Renyi entropy o=4	0.005	0.344	0.9948	0.6557
IDE ($S_{FDE}=8$)	0.38	0.768	0.6135	0.2318
IDE ($S_{FDE}=9$)	0.28	0.793	0.7198	0.2068
AIDE	0.2755	0.786	0.7245	0.2135
IFDE ($S_{FDE}=8$)	0.397	0.838	0.6026	0.1615
IFDE ($S_{FDE}=9$)	0.421	0.824	0.5781	0.1745
AIFDE	0.4828	0.826	0.5172	0.174
TDL-D				
ED	0.013	0.232	0.988	0.768
Shannon entropy	0.010	0.366	0.990	0.634
Renyi entropy o=2	0.007	0.309	0.993	0.691
Renyi entropy o=3	0.003	0.216	0.997	0.784
Renyi entropy o=4	0.006	0.207	0.994	0.793
IDE ($S_{FDE}=8$)	0.180	0.535	0.820	0.465
IDE ($S_{FDE}=9$)	0.226	0.494	0.774	0.506
AIDE	0.209	0.485	0.791	0.515
IFDE ($S_{FDE}=8$)	0.226	0.561	0.774	0.439
IFDE ($S_{FDE}=9$)	0.197	0.513	0.803	0.488
AIFDE	0.221	0.528	0.779	0.472
TDL-E				
ED	0.010	0.105	0.990	0.895
Shannon entropy	0.021	0.488	0.979	0.513
Renyi entropy o=2	0.019	0.481	0.981	0.519
Renyi entropy o=3	0.004	0.412	0.996	0.588
Renyi entropy o=4	0.005	0.419	0.995	0.581
IDE ($S_{FDE}=8$)	0.336	0.667	0.664	0.333
IDE ($S_{FDE}=9$)	0.262	0.694	0.738	0.306
AIDE	0.233	0.689	0.767	0.311
IFDE ($S_{FDE}=8$)	0.392	0.682	0.608	0.318
IFDE ($S_{FDE}=9$)	0.357	0.731	0.643	0.269
AIFDE	0.368	0.737	0.632	0.263

## Data Availability

The data presented in this study are available on request from the corresponding author. The data are not publicly available due to the not finalized publication process on the JRC open data web site.

## Sample Availability

The data presented in this study are available on request from the corresponding author. The data are not publicly available due to the not finalized publication process on the JRC open data web site.

## Abbreviations

The following abbreviations are used in this manuscript:

AIFDE	Adaptive Improved Fluctuation Dispersion Entropy
AIDE	Adaptive Improved Dispersion Entropy
AWGN	Additive White Gaussian Noise
DE	Dispersion Entropy
DT	Decision Tree
IFDE	Improved Fluctuation Dispersion Entropy
IDE	Improved Dispersion Entropy
KNN	K Nearest Neighbor
NCDF	Normal Cumulative Distribution Function
PRF	Pulse Repetition Frequency
RBF	Radial Basis Function
RE	Renyi Entropy
SE	Shannon Entropy
SNR	Signal to Noise Ratio
SVM	Support Vector Machine
TDL	Tapped Delay Line
